# Towards the diversification of lactococcal starter and non‐starter species in mesophilic dairy culture systems

**DOI:** 10.1111/1751-7915.14320

**Published:** 2023-07-15

**Authors:** Jennifer Mahony, Francesca Bottacini, Douwe van Sinderen

**Affiliations:** ^1^ School of Microbiology University College Cork Cork Ireland; ^2^ APC Microbiome Ireland University College Cork Cork Ireland; ^3^ Department of Biological Sciences Munster Technological University Cork Ireland

## Abstract

*Lactococcus* is one of the earliest identified fermentative bacterial genera and among its member species, the dairy‐associated *Lactococcus lactis* and *Lactococcus cremoris* are undoubtedly the best studied. These two species are believed to have evolved from plant‐associated lactococci and through genome decay and acquisition of plasmids, have adapted to the dairy niche. The past decade has witnessed a surge of activity in novel lactococcal species identification from insect, plant and animal sources. Currently, 22 *Lactococcus* species are described and in this review, we summarise the genome characteristics of and phylogenetic relationships among these species. Furthermore, we explore the role of mobile elements including plasmids and bacteriophages in the diversification of lactococcal species. The pace of identification of novel lactococcal species suggests that the number of lactococcal species is likely to continue to grow. With additional sequence data for the emerging species, it will be possible to perform pathogenicity/virulence risk evaluations and generate extensive insights into the niche adaptation strategies through which they have evolved.

## Graphical abstract

Phylogenomic analysis of 22 *Lactococcus* species. Lactococcal species are associaetd with diverse ecological niches including dairy, plants and insects.

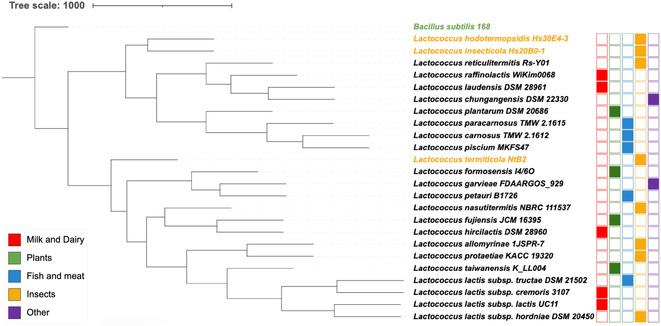

## INTRODUCTION

Mesophilic dairy starter culture systems are largely reliant on strains of *Lactococcus cremoris* and/or *Lactococcus lactis*. The number and type of strains employed in a given dairy fermentation practice may be defined or undefined. Furthermore, the production of certain regional cheeses and fermented dairy products rely on the autochthonous microbiota of the milk substrate and/or the fermentation vessel to initiate the fermentation process. In defined starter cultures the exact identity, number and characteristics of the strains used are known and such starter culture systems are widely applied in the production of Cheddar‐style cheeses globally (Poudel et al., 2022). In contrast, the production of many Italian‐ and Dutch‐style cheeses is achieved through the use of mixed starter cultures or traditional production regimes, which are compositionally diverse and incorporate multiple bacterial species and, in some cases, fungal species as well (Frantzen et al., 2018). While the majority of these mesophilic artisanal or traditional mixed starter cultures are dominated by *Lactococcus cremoris* and/or *L. lactis*, additional starter or non‐starter microbial community members contribute to the organoleptic properties of the individual final products. In yoghurt production, *Streptococcus thermophilus* and *Lactobacillus delbrueckii* subsp. *bulgaricus* are well known for their proto‐cooperative behaviour to accelerate the acidification of the milk substrate (Yamauchi et al., 2019). In this relationship, *S. thermophilus* produces formate, a metabolite required by *Lb. bulgaricus* to grow, while in return the activity of cell wall‐bound proteases of *Lb. bulgaricus* supports the growth of the streptococcal strain (Liu et al., 2009). Such proto‐cooperative relationships are less well‐defined in mesophilic cultures; however, it is likely that mutualistic relationships exist within mixed starter culture systems. Mesophilic starter cultures are classified in the dairy industry as D‐, L‐, DL‐ or O‐cultures based on the application of *L. lactis* biovar diacetylactis (D‐), *Leuconostoc* (L‐) or a combination of both species (DL‐) for flavour formation in addition to *Lactococcus lactis* strain(s) that are the major contributors to acid formation. O‐cultures are those starter cultures which solely rely on *L. lactis* for acidification. This traditional classification of defined culture systems may now be challenged by emerging reports of novel lactococcal species that are capable of growth in milk and that may support or enhance dairy fermentations as a starter or adjunct cultures.

This review explores the current state of play in artisanal and mixed starter cultures to highlight emerging lactococcal species that may play a role in culture performance and functional attributes of fermented dairy products. Awareness of the emerging complementary species in these systems may enhance strategies to preserve and bolster the robustness of both defined and undefined starter culture systems and improve the sustainability of food fermentations through a reduction in food waste.

## *LACTOCOCCUS*: A DIVERSE GENUS

*Lactococcus* was discovered through the first studies of fermentation processes by Joseph Lister in the late 1800's at which point it was termed *Bacterium lactis* and was later renamed *Streptococcus lactis* by Orla Jensen. These early studies of fermentation processes and dairy starter cultures classified bacteria based on their growth capabilities and characteristics. Owing to their industrial relevance, lactococcal research efforts have focused almost exclusively on the earliest described members of the genus (Konkit et al., 2014), that is, *L. lactis* and *L. cremoris*. *L. cremoris* was originally classified as a subspecies of *L. lactis* but has recently been elevated to the species level based on sequence comparisons of the 16S rRNA gene, *rpoB*, *recA* and *pheS* genes (Li et al., 2019). While multi‐locus sequence typing is appropriate to differentiate at the species level, it is unable to distinguish strains within a given species. However, at the species level, the current classification aligns well with the overall technological properties of members of these two species. For example, strains of *L. lactis* can tolerate up to 4% sodium chloride, temperatures of up to 40°C and are primarily applied for their fast growth and acidification properties. In contrast, *L. cremoris* strains typically exhibit a lower salt and temperature tolerance, often display slower growth profiles and are associated with flavour development properties (Obis et al., 2001).

In recent years, a number of reports of novel lactococcal species have established that this genus is quite diverse and its member species are present in a wide range of ecological niches (Goodman et al., 2017; Heo et al., 2019, 2020; Hilgarth et al., 2020; Noda et al., 2018, 2020) (Table 1). Currently (as of March, 2023) 22 species of *Lactococcus* are described in the NCBI taxonomy browser with 694 genome sequences available in the database (Table 1). Among the described lactococcal species, members of five species have been isolated from animal milk, that is, *L. lactis*, *L. cremoris*, *Lactococcus raffinolactis*, *Lactococcus laudensis* and *Lactococcus hircilactis*. Perhaps unsurprisingly, the genomes of 367 and 111 *L. lactis* and *L. cremoris*, respectively, represent the majority of the currently available genome data for this genus (as of March, 2023). Six lactococcal species members were isolated from the gut of insects including termites and beetles, five species were originally isolated from animal or fish sources and four species were described to be associated with vegetable plant material (Table 1). Average genome sizes range from 1.7 Mb (*L. termiticola*) to 2.8 Mb (*L. allomyrinae*). The dairy species *L. lactis* and *L. cremoris* genomes are typically at the higher end of this range with chromosomes of approximately 2.5 Mb in length (excluding plasmid content). The emerging dairy species, *L. hircilactis*, *L. laudensis* and *L. raffinolactis* have average genome sizes of 2.6, 2.3 and 2.3 Mb, respectively (Table 1).

**TABLE 1:** Genome characteristics and sources of the 22 currently known lactococcal species.

*Lactococcus* species	Isolation source	#genome sequences available	Average genome size (Mb)	#CDS	Reference of early descriptions of the species
*Allomyrinae*	Gut of *Allomyrina dichotoma* larva (beetle)	1	2.8	2665	Heo et al. (2019)
*Carnosus*	Red meat	15	2.2	2102	Hilgarth et al. (2020)
*Chungangensis*	Activated sludge	4	2.1	2214	Cho et al. (2008)
*Cremoris*	Dairy	111	2.5	2631	Li et al. (2019)
*Formosensis*	Fermented broccoli stems	9	2.2	2201	Chen et al. (2014)
*Fujiensis*	Chinese cabbage leaves	2	2.1	2289	Cai et al. (2011)
*Garvieae*	Bovine mastitis; fish pathogen	75	2.1	2124	Collins et al. (1983)
*Hircilactis*	Goat milk	1	2.6	2580	Meucci et al. (2015)
*Hodotermopsidis*	Gut of *Hodotermopsis sjostedti* (termite)	1	2.3	2233	Noda et al. (2020)
*Insecticola*	Gut of *Hodotermopsis sjostedti* (termite)	1	2.0	1926	Noda et al. (2020)
*Lactis*	Dairy, plant	367	2.5	2613	Tan‐a‐ram et al. (2011)
*Laudensis*	Goat milk	2	2.3	2334	Meucci et al. (2015)
*Nasutitermitis*	Gut of *Nasutitermes hainanensis* (termite)	1	2.2	2154	Yan Yang et al. (2016)
*Paracarnosus*	Red meat	5	2.2	2194	Hilgarth et al. (2020)
*Petauri*	Facial abscess of a sugar glider (marsupial) – *Petaurus breviceps; fish pathogen*	68	2.1	2088	Egger et al. (2023); Goodman et al. (2017)
*Piscium*	Salmonid fish	6	2.3	2292	Williams et al. (1990)
*Plantarum*	Frozen peas	2	2.0	1902	Collins et al. (1983)
*Protaetiae*	Gut of *Protaetia brevitarsis seulensis* larva (beetle)	1	2.7	2611	Heo et al. (2020)
*Raffinolactis*	Milk, dairy	16	2.3	2343	Klijn et al. (1995)
*Reticulitermitis*	Gut of *Reticulitermes speratus* (termite)	1	2.2	2168	Yuki et al. (2018)
*Taiwanensis*	Fresh cummingcordia	4	1.9	1964	Chen et al. (2013)
*Termiticola*	Gut of *Nasutitermes takasagoensis* (termite)	1	1.7	1694	Noda et al. (2018)

**FIGURE 1:**
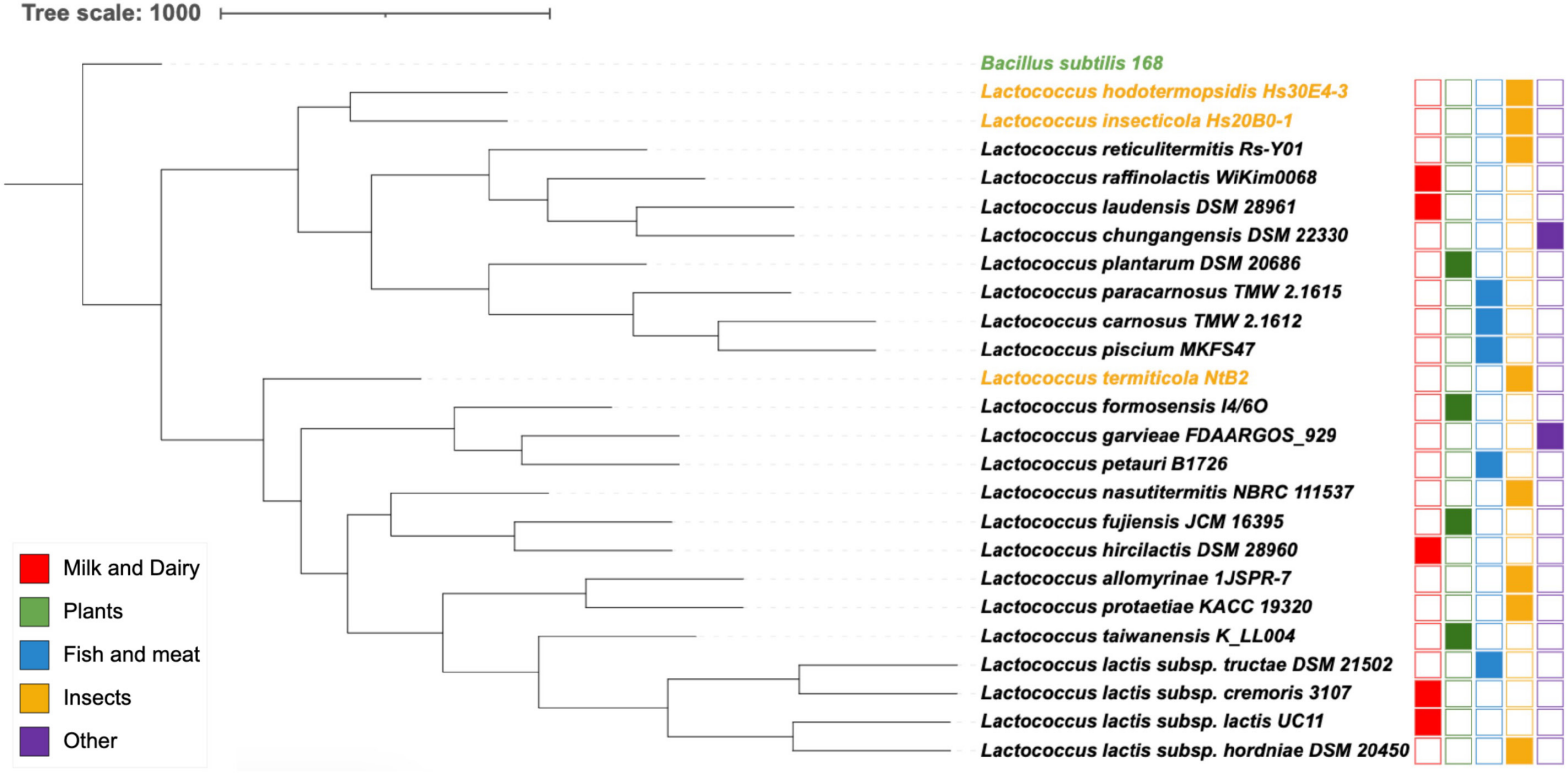
Phylogenomic analysis of *Lactococcus* species. Unrooted supertree of the *Lactococcus* genus. Phylogenomic analysis was performed based on nucleotide alignments of 478 single‐copy orthologues identified between *Lactococcus*‐type strains and the outgroup *Bacillus subtilis* (green). Orthologous and single‐copy genes were obtained using OrthoFinder (Emms & Kelly, 2019) and the amino acid sequence of the strain of interest obtained from the NCBI RefSeq database. Multiple sequence alignment of protein sequences in each single‐copy orthogroup was conducted using MUSCLE v.3.8.31 (Edgar, 2004), followed by alignment refinement using Gblocks (Talavera & Castresana, 2007) and the construction of a maximum likelihood phylogenetic tree using PhyML v3.0 (Guindon et al., 2010), followed by concatenation of the tree. A final consensus tree was computed using the Consense module from Phylip package v3.69 (http://evolution.genetics.washington.edu/phylip.html) using the majority rule method. The webserver iTOL was used for tree visualisation (https://itol.embl.de/). A list of computed single‐copy orthologues is provided in Table S1.

Based on phylogenetic analysis of 478 single‐copy core genes across representatives of the 22 species of *Lactococcus*, it appears that members of this genus do not necessarily cluster based on their origin of isolation. However, phylogenetic analysis of members of this genus clearly identifies two distinct clades, both containing insect isolates as possible ancestors. While plant material is considered the natural habitat of *Lactococcus* strains (Laroute et al., 2017), one can postulate that this genus has originated from two distinct lineages, which should be further studied based on their metabolic abilities. It is noteworthy that “domesticated” and “environmental” species distribute across the entire phylogenetic tree, suggesting a common origin (Figure 1). Furthermore, in line with a previous report (Pérez et al., 2011), the historic *Lactococcus lactis* subspecies are organised into two distinct lineages, one containing *L. lactis* subsp. *lactis* and the other *L. lactis* subsp. *cremoris* justifying their recent separation as distinct species (Li et al., 2019).

## NON‐STARTER LACTOCOCCI: OPPORTUNITY OR RISK?

While the functionality of the dairy‐associated species *L. raffinolactis*, *L. laudensis* and *L. hircilactis* in dairy fermentations is not well defined, there is evidence to suggest that members of these species have acquired the ability to grow in milk. For example, *L. raffinolactis* strain 4877 is described to possess the complete set of genes required for lactose fermentation as well as those involved in oligopeptide transport (Meslier et al., 2012). This strain was isolated from a natural dairy starter culture in France and while it is described as a non‐starter component of the culture, it was present in sufficient abundance to be distinguished from the dominant starter bacterial species within the culture. *L. raffinolactis* strain 37 was isolated from raw cow's milk and its growth and acid production were stimulated by the metabolites (in the cell‐free supernatant) of a *L. lactis* strain (Kimoto‐Nira et al., 2012). *L. raffinolactis* strains are incapable of growth at 40°C and are sensitive to the presence of 4% sodium chloride in the growth medium. Strains of this species do not perform arginine deamination and require supplementation with yeast extract to clot milk in pure culture (Kimoto‐Nira et al., 2012). Interestingly, these characteristics are reminiscent of *L. cremoris* strains with the exception of growth in milk without supplementation. Therefore, while strains of *L. raffinolactis* are unlikely to be employed as starter cultures in the dairy industry, they may be useful adjunct cultures to support or enhance the acidification and/or flavour profiles of mesophilic starter culture systems.

*L. hircilactis* and *L. laudensis* have similarly been evaluated for their ability to acidify milk and contribute to cheese‐making processes (Tidona et al., 2018). Strains of both species were observed to grow in milk achieving a pH of 4.8 and 5.5 after 24 h at 30°C, respectively. Furthermore, when strains of these species were added as adjuncts to a commercial starter culture, distinct flavour profiles were observed in the final products highlighting their active contribution to the fermentation process and organoleptic properties of the food (Tidona et al., 2018).

Beyond dairy‐associated species, non‐dairy species are also being explored for their potential applicability to dairy fermentations. For example, *L. chungangensis* CAU28 is capable of degrading casein and also possesses aminohydrolase, which is associated with flavour formation (Konkit et al., 2014). Comparison of the growth characteristics of *L. chungangensis* CAU28 and *L. lactis* strain 2769 in 5% and 20% skim milk established that the growth capabilities of both strains were similar highlighting the potential of *L. chungangensis* to adapt to dairy fermentations. Additionally, *L. chungangensis* CAU28 exhibits aldehyde dehydrogenase activity, converting acetaldehyde to acetic acid. In murine trials, the addition of the strain to cream cheese that was fed to mice was associated with an increase in short‐chain fatty acid production supporting its application in functional foods (Kim et al., 2019; Konkit et al., 2016).

A recent survey of Chinese dairy products identified *L. petauri* in raw buffalo milk, fermented buffalo milk and the area around the buffalo udder (Huang et al., 2021). Similarly, *L. petauri* strains were isolated from traditional Montenegrin brine cheeses and the genome of *L. petauri* INF110 was found to harbour the antibiotic resistance‐associated genes, *mdtA* and *clpI* (Martinovic et al., 2021). The genome of *L. petauri* B1726 harbours adhesion‐associated genes as well as genes that encode functions that have been claimed to facilitate immune system evasion including phosphoglucomutase and D‐alanine‐D‐alanyl carrier protein ligase although further investigation would be required to substantiate this claim (Desiderato et al., 2022). Haemolytic activity was also observed on blood agar plates demonstrating the pathogenic potential of strains of this species and thus its poor suitability for application in food systems. Furthermore, the first descriptions of this species are associated with animal (marsupial and fish) pathogenesis (Egger et al., 2023; Goodman et al., 2017). Therefore, it is prudent to evaluate the genomes of emerging lactococcal species to evaluate their safety and appropriateness for deliberate application in food fermentations. Currently, the genomes of 68 *L. petauri* strains are available rendering it timely for a species‐wide evaluation of the threat posed by members of this species to human and animal health through its presence in foods and food production environments. Strains of both *L. garvieae* and *L. petauri* are reported to produce the heat‐stable, non‐lantibiotic bacteriocin, garvicin Q, which elicits antimicrobial activity against *Listeria*, *Carnobacterium*, *Enterococcus*, *Lactococcus*, *Leuconstoc* and certain lactobacilli and pediococci (Tymoszewska et al., 2017). Strains of *Pediococcus acidilactici* and *Lactobacillus kunkeei* were observed to be resistant to garvicin Q as were pathogenic species of *Campylobacter*, *Streptococcus*, *Bacillus* and *Staphylococcus*. Combined, these data demonstrate the antagonistic activity of garvicin Q against several industrially significant and/or commensal lactic acid bacterial genera with just limited impact on several pathogenic genera.

## THE ROLE OF MOBILE ELEMENTS IN GENOME DIVERSIFICATION AND NICHE ADAPTATION

Adaptation to the dairy niche has culminated in considerable genome decay in *L. cremoris* and *L. lactis* (Kelleher et al., 2017). In parallel, members of these species have acquired plasmids to optimise their adaptation to the dairy substrate. These plasmids harbour genes associated with lactose metabolism, citrate metabolism, casein degradation, peptide transport and bacteriophage resistance (for an extensive review of this see Kelleher et al., 2017). Strains of *L. lactis* and *L. cremoris* are reported to harbour up to 12 plasmids (van Mastrigt et al., 2018). Additionally, certain *L. cremoris* strains are also described to harbour megaplasmids (i.e. those >100 kb) and the plasmidome of *L. lactis* and *L. cremoris* strains can account for up to 10% of the overall genome content of a given strain (Kelleher et al., 2019).

Based on a search of plasmid sequences associated with the 22 lactococcal species in the NCBI database, six species are described to harbour plasmids in addition to *L. cremoris* and *L. lactis*, that is, *L. allomyrinae*, *L. formosensis*, *L. garvieae*, *L. petauri*, *L. piscium* and *L. raffinolactis*. The genome of the single sequenced isolate of *L. allomyrinae* harbours two plasmids (8331 and 54,925 bp). The eight strains of *L. formosensis* for which genome sequence data are available are observed to harbour up to five plasmids ranging in size from 3771 to 35,568 bp. *L. garvieae* strains harbour up to four plasmids ranging in size from 3686 to 85,819 bp. *L. petauri* and *L. piscium* strains harbour up to three plasmids and *L. raffinolactis* harbour up to six plasmids (2725–82,612 bp). Plasmids of *L. petauri* and *L. garviae* possess relaxase‐encoding genes, which may signify a role in conjugation and/or mobilisation of DNA between strains of these species. Dairy lactococci have been described to employ conjugation to transfer genetic material, while non‐conjugative plasmids of *L. lactis* and *L. cremoris* have also been observed to co‐mobilise along with a conjugatable plasmid (Ortiz Charneco et al., 2021). Furthermore, discrete nanotube‐like structures have been observed in lactic acid bacteria including *L. cremoris* and *S. thermophilus*. These nanotube structures have been associated with the non‐conjugative transfer of plasmid DNA transfer within and between bacterial species (Morawska & Kuipers, 2023). Furthermore, it was demonstrated that DNA transfer could be mediated between distant bacterial genera, that is, plasmid DNA was transferred between strains of *Bacillus subtilis* and *S. thermophilus* or *L. cremoris*. Therefore, it is conceivable that plasmids of other lactococcal species are either conjugative, co‐mobilisable or transferrable via nanotubes and contribute to niche adaptation as has been described in *L. cremoris* and *L. lactis*.

## BACTERIOPHAGE GENOME SEQUENCE SIMILARITIES SUGGEST COMMON ANCESTRY

Bacteriophages (or phages) are bacterial viruses that specifically infect strains of a given bacterial species. Phages may follow one of two dominant cycles, that is, the lytic or lysogenic cycle. In the lytic cycle, the phage adsorbs to its host via a suitable and specific cell surface receptor. The phage subsequently injects its genome into the host cell cytoplasm where it replicates, lyses the host cell and releases progeny phages into the surrounding environment. In the lysogenic cycle, the phage adsorbs to the host cell surface receptor and injects its genome into the cytoplasm similar to the lytic cycle. Following this, the phage genome becomes incorporated into the host chromosome via attachment sites present in the host chromosome (attB) and complementary to homologous sequences at the phage genome termini (attP). In the integrated state, the phage is termed a prophage and it replicates in situ along with the host chromosome. Under certain conditions, the prophage may be induced from the host chromosome and will complete the lytic cycle, producing progeny phage particles. In the prophage‐inducing state, dairy lactococci have also been shown to exhibit increased extracellular vesicle formation through the activity of the phage holin‐lysin system (Liu et al., 2022). This phenomenon is not confined to lactococci and is also known to occur in a wide range of Gram‐positive and Gram‐negative bacteria (Dean et al., 2022). Extracellular vesicles may carry nucleic acids, viral particles and enzymes within their membranes and they are implicated in functions including cell‐to‐cell communication, elimination of competitors and nutrient sensing (Bose et al., 2020; Liu et al., 2022). The chromosomes of *L. lactis* and *L. cremoris* strains are reported to harbour up to six prophages all of which belong to the heterogenous P335 phage group. In cheese‐making, prophage induction is a potential risk to the fermentation process as cells may lyse before acidification is complete. However, lysis of a sub‐population of cells may also be considered beneficial through the release of intracellular flavour‐associated enzymes. Perhaps, extracellular vesicle production may equally be considered beneficial through the delivery of such enzymes to the extracellular environment. Additionally, extracellular vesicles carrying nucleic acids may act as vehicles for intra‐ or interspecies transfer of genetic material (Tran & Boedicker, 2019). The inter‐species transfer of plasmids has been shown to be origin‐specific and dependent on the copy number of the plasmid, with an increased transfer of high copy number plasmids (Tran & Boedicker, 2019). Therefore, as additional information regarding the prophages and plasmids of emerging lactococcal species becomes available, studies relating to vesicle‐mediated transfer of genetic material between diverse lactococcal species will undoubtedly enhance strain development efforts.

In addition to prophages, there are nine virulent lactococcal phage species that infect *L. lactis* and/or *L. cremoris* strains (Deveau et al., 2006). Among these, member of the *Ceduovirus*, *Skunavirus* and P335 species are the most frequently encountered in dairy fermentation environments. Sequence comparisons of *L. garvieae* prophages established that several of them share sequence similarity with P335 phages of *L. lactis/cremoris* and suggest that these phages may have originated from the same ancestor (Eraclio et al., 2017). Interestingly, the virulent *L. garvieae* phage GE1 harbours significant sequence identity to ceduoviruses (Eraclio et al., 2015). Despite the sequence similarity, phage GE1 was unable to infect any of the 58 dairy lactococcal strains tested (Eraclio et al., 2015). Conversely, skunaviruses (which infect *L. lactis* diaectylactis) isolated from Polish dairy whey samples were evaluated for their ability to infect not only *L. lactis* and *L. cremoris* strains but also *L*. *garvieae*, *L*. *plantarum*, *L*. *raffinolactis* or *L*. *laudensis* strains (Chmielewska‐Jeznach et al., 2018). These studies are remarkable in exploring the cross‐infection potential of dairy and non‐dairy lactococcal phages and provide a noteworthy foundation for future studies to define cross‐species relationships or receptor binding potential.

The genome sequences of the virulent lactococcal *Teubervirus* (formerly termed the P087 phages) and *Fremauxvirus* (formerly termed the 1706 phages) members bear similarities to those of prophages of other *Bacillota* genera (Garneau et al., 2008). Furthermore, the dairy streptococcal 987 group phage genomes exhibit significant sequence similarity to those of lactococcal P335 phages including ul36 (McDonnell et al., 2016). The sequence similarity shared by dairy and non‐dairy lactococcal phages and beyond signifies the extensive genetic transfer and genome plasticity of phages. While phage‐host interactions of dairy lactococci are well studied, those of other lactococcal species have not yet been defined. As increasing numbers of isolates and associated genome sequences of the emerging species become available, it will be possible to establish the prevalence and diversity of prophages as well as virulent phages that infect these species.

## PREVALENCE AND DIVERSITY OF CRISPR‐CAS SYSTEMS IN LACTOCOCCAL SPECIES

*L. lactis* and *L. cremoris* are reported to harbour a diverse array of anti‐phage systems including restriction/modification, abortive infection and superinfection exclusion systems (Chopin et al., 2005; Mahony et al., 2008; Malesevic et al., 2021). These frequently stacked systems provide a series of barriers to phage proliferation in dairy fermentations. Typically, dairy lactococci do not rely on clustered regularly interspaced palindromic repeat (CRISPR) systems and very limited numbers of strains are reported to harbour them (Millen et al., 2019). The first among these to be identified was a Type III‐A CRISPR‐Cas system that was identified on a conjugative plasmid in an *L. lactis* strain (Millen et al., 2012). Beyond dairy lactococci, it is not clear if other species harbour CRISPR‐Cas (CRISPR‐associated genes) systems or if they also rely on the protection by alternative anti‐phage systems. To evaluate this, the CRISPRCasFinder database was searched for CRISPR‐Cas systems using the search term *Lactococcus* and selecting evidence levels 2, 3 and 4, which represent higher confidence levels. Using this approach, candidate CRISPR‐Cas systems were identified in the genomes of *L. piscium*, *L. raffinolactis*, *L. protaetieae*, *L. allomyrinae*, *L. carnosus* and *L. paracarnosus* strains (Table 2). These instances of CRISPR‐Cas systems represent eight out of 87 lactococcal genomes evaluated in the CRISPRCasFinder database highlighting the relatively low incidence of these defence mechanisms in *Lactococcus* as a genus and suggesting a reliance on alternative resistance mechanisms similar to dairy lactococci (https://crisprcas.i2bc.paris‐saclay.fr/MainDb/StrainList/Lactococcus). Representatives of Type I‐E, II‐A, II‐C and III‐A CRISPR‐Cas systems were identified in eight lactococcal strains with six of the eight strains harbouring a single such system (Table 2). The two exceptions to this observation were two *L. raffinolactis* strains whose genomes both harbour three CRISPR‐Cas systems (Table 2). Most of the identified systems harbour a significant number of spacers suggesting that they are likely functional. While this data represent a subset of approximately 12% of the total number of sequenced lactococcal genomes, it provides an insight into the distinct anti‐phage strategies employed among the different lactococcal species. It is tempting to speculate that the CRISPR‐Cas systems identified in dairy lactococci have been acquired from non‐starter lactococcal species such as *L. raffinolactis*.

**TABLE 2:** Summary of the lactococcal CRISPR‐Cas systems identified by CRISPRCasFinder including the type of system and the number of spacers within the array of each system.

Strain name	Type I‐E	Type II‐A	Type II‐C	Type III‐A
*L. piscium* CMTALT02	**61**			
*L. raffinolactis* Lr_18_12S		**34**		
*L. raffinolactis* Lr_19_14	**24**	**41**		**15**
*L. raffinolactis* Lr_19_5		**55**	**9**	**14**
*L. allomyrinae* 1JSPR‐7			**41**	
*L. protaetiae* KACC 19320			**40**	
*L. carnosus* TMW 2.1612		**26**		
*L. paracarnosus* TMW 2.1615	**65**			

*Note*: Blue = absent; Pink = present (#spacers).

## DETECTION AND CLASSIFICATION OF LACTOCOCCI

Dairy lactococci were traditionally classified based on their phenotypic attributes including salt and thermal tolerance as outlined above. As awareness of the diversity of dairy lactococcal strains increased, rapid identification tools were developed that are largely PCR‐based (Garde et al., 1999; Khemariya et al., 2013; Mahony et al., 2013; Odamaki et al., 2011). The emergence of a novel species of lactococcal species also prompted the development of a multiplex PCR system for the identification and classification of isolates of the seven species of *Lactococcus* that were described at that time, that is, *L. garvieae*, *L. piscium*, *L. plantarum*, *L. raffinolactis*, *L. chungangensis*, *L. lactis* and *L. fujiensis* (Odamaki et al., 2011). While 15 additional species of *Lactococcus* are now known to exist, this PCR system continues to have relevance in the dairy industry as many of the primary starter and non‐starter dairy species can be detected by this PCR system. Future incarnations of this PCR system could include primers for the detection of *L. cremoris*, *L. laudensis* and *L. hircilactis* to identify the presence of strains of such species in artisanal and mixed starter culture systems.

While the phenotypic and genome characterisation of most of the recently identified lactococcal species is quite limited due to the low number of reported isolates, it is important to understand the metabolic potential of these species to establish their likely adaptation to different ecological niches. A pertinent example is the carbohydrate utilisation profile of strains of lactococcal species. This is particularly well‐described for the dairy lactococcal species *L. lactis* and *L. cremoris* based on both genome sequence analysis and phenotypic characterisation and is, perhaps unsurprisingly, largely focused on lactose metabolism. Within these species, however, are strains that appear to have adapted broader carbohydrate utilisation capabilities including *L. lactis* strain A12, which was isolated from sourdough (Passerini et al., 2013). Genome comparisons with the model *L. lactis* strain IL1403 identified unique gene content representing 23% of the *L. lactis* A12 genome. Phenotypic evaluation established that this strain was capable of metabolising various plant‐derived carbohydrates including cellobiose, arabinose, raffinose and trehalose while it is incapable of growth on lactose. Therefore, it is important to note that while the majority of *L. lactis* strains are dairy‐associated, there are plant‐derived isolates with highly distinct metabolic profiles, which is likely responsible for the observed genetic variability within this species. *L. raffinolactis* strains are capable of fermenting α‐galactosides including raffinose and melibiose and the resulting α‐galactose is further degraded through either the tagatose 6‐phosphate or the Leloir pathway (Boucher et al., 2003). *L. hircilactis* acidifies milk well and is capable of fermenting galactose, lactose, melibiose, raffinose, sucrose, mannitol, gentibiose and starch, whereas *L. laudensis* exhibits limited milk acidification yet is able to ferment lactose, galactose, sucrose, mannitol and xylose (Meucci et al., 2015).

A recent genome analysis of *L. petauri* CF11 identified 137 genes predicted to be associated with carbohydrate transport and metabolism (Ou et al., 2020) including those associated with the transport of cellobiose, fructose, galactitol, lactose, mannose, sucrose, trehalose, mannitol and maltose. *L. piscium*, a food spoilage psychrotroph, is capable of fermenting a wide range of carbohydrates including glucose, fructose, lactose, galactose, gluconate, gentiobiose, mannose, maltose, melibiose, trehalose, arbutin, l‐arabinose, *N*‐acetylglucosamine, salicin and d‐raffinose (Saraoui et al., 2016). Such fermentation profiles are useful to establish the likely natural source of these organisms as well as to predict their ability to adapt to other ecological niches. As increasing numbers of isolates of lactococcal species become available (and their associated genome sequences), it will be possible to generate a broad view of their metabolic abilities and corresponding adaptability. At present, it is tempting to speculate that most of the non‐dairy derived species would have a broader carbohydrate metabolic capability while that of dairy strains has been reduced significantly through adaptation to the milk substrate.

## CONCLUSIONS AND FUTURE PERSPECTIVES

Currently, 22 species of *Lactococcus* are defined; however, given the recent surge in novel species identification, it seems likely that many more will be identified in the coming decade. Furthermore, with increasing numbers of isolates of each species, it will be possible to evaluate the true extent of genetic and metabolic diversity of these organisms. Genome analysis of (pro)phages of the dominantly studied lactococcal species including *L. lactis*, *L. cremoris* and *L. garvieae* suggest a common ancestor for these phages and likely their hosts. Reports of lactococcal phages are largely confined to these species thusfar; however, it is likely that phages will be isolated for other lactococcal species in the near future. The comparison of virulent or temperate phages of additional lactococcal species would provide significant insights into their history and evolutionary pathways. Therefore, while the last decade has seen a massive surge in interest in establishing the diverse nature of the *Lactococcus* genus, it is clear that we have only begun to scratch the surface pertaining to the extent and nature of lactococcal diversity.

## AUTHOR CONTRIBUTIONS

**Jennifer Mahony:** Conceptualization (equal); funding acquisition (equal); investigation (equal); resources (equal); writing – original draft (equal); writing – review and editing (equal). **Francesca Bottacini:** Conceptualization (equal); data curation (equal); formal analysis (equal); investigation (equal); resources (equal); writing – original draft (equal); writing – review and editing (equal). **Douwe van Sinderen:** Conceptualization (equal); funding acquisition (equal); writing – review and editing (equal).

## CONFLICT OF INTEREST STATEMENT

The authors have no conflict of interest.

## Supporting information


Table S1.
